# Enhanced Distributed Energy-Efficient Clustering (DEEC) Protocol for Wireless Sensor Networks: A Modular Implementation and Performance Analysis

**DOI:** 10.3390/s25134015

**Published:** 2025-06-27

**Authors:** Abdulla Juwaied, Lidia Jackowska-Strumillo, Michal Majchrowicz

**Affiliations:** Institute of Applied Computer Science, Lodz University of Technology, ul. Stefanowskiego 18, 90-537 Lodz, Poland; lidia.jackowska-strumillo@p.lodz.pl (L.J.-S.); michal.majchrowicz@p.lodz.pl (M.M.)

**Keywords:** wireless sensor networks, distributed energy-efficient clustering, sensor nodes, base station, energy

## Abstract

Wireless Sensor Networks (WSNs) are a component of various applications, including environmental monitoring and the Internet of Things (IoT). Energy efficiency is one of the significant issues in WSNs, since sensor nodes are usually battery-powered and have limited energy resources. The Enhanced Distributed Energy-Efficient Clustering (DEEC) protocol is one of the most common methods for improving energy efficiency and network lifespan by selecting cluster heads according to residual energy. Nevertheless, standard DEEC methods are limited in dynamic environments because of their fixed nature. This paper presents a novel modular implementation of the DEEC protocol for Wireless Sensor Networks, addressing the limitations of the standard DEEC in dynamic and heterogeneous environments. Unlike the typical DEEC protocol, the proposed approach incorporates realistic energy models, supports heterogeneous nodes, implements load balancing, and enables dynamic cluster head selection Numerical simulations in MATLAB^®^ demonstrate that the improved DEEC protocol achieves a 133% longer stability period (first node death at 1166 rounds vs. 472 rounds), nearly doubles the network lifetime (4000 rounds vs. 2111 rounds), and significantly enhances energy efficiency compared to the standard DEEC. These results verify the effectiveness of the proposed enhancements, making the protocol a robust solution for modern WSN and IoT applications.

## 1. Introduction

Wireless Sensor Networks (WSNs) comprise multiple sensor nodes deployed to sense and report data from varying environments. WSNs form the core technology used in such applications as environmental monitoring, health, and smart cities. These networks consist of spatially distributed sensor nodes that autonomously sense, process, and transmit data to a central base station. WSNs face significant challenges despite their transformative potential, particularly in resource-constrained environments. The most critical of these is energy efficiency, as sensor nodes are typically battery-powered and often deployed in locations where battery replacement is impractical. Prolonging network lifetime while maintaining reliable communication and data integrity is a primary research focus. Scalability is another challenge, as increasing the number of nodes complicates communication and data aggregation, reliability in the face of node failures and environmental interference, and security, given the vulnerability of wireless communication to eavesdropping and attacks. Addressing the previous challenges is essential for the practical deployment and sustainability of WSNs [1,2,3,4].

To meet these demands, researchers have developed a variety of energy-efficient communication protocols. Among them, clustering-based protocols have proven especially effective. In these protocols, nodes are grouped into clusters, with each managed by a cluster head (CH) responsible for aggregating and forwarding data to the base station. The DEEC (Distributed Energy-Efficient Clustering) protocol is a prominent protocol designed to improve energy efficiency and extend the network lifetime. DEEC’s adaptive mechanism ensures that nodes with higher energy are more likely to become CHs, distributing the energy load more evenly and delaying node death [3,4].

However, the original DEEC protocol has limitations, particularly in heterogeneous networks where nodes may have different initial energy levels. It does not fully account for advanced or super nodes with greater energy reserves nor address issues such as load balancing among clusters, packet loss, or data aggregation at CHs. These shortcomings can lead to suboptimal energy usage, reduced network stability, and shorter operational lifespans. Several enhancements to DEEC have been proposed to overcome these issues, such as introducing probabilistic CH selection, load balancing mechanisms, and support for heterogeneous node energy levels [5]. The present study focuses on developing and evaluating an improved DEEC protocol that incorporates a more realistic energy model, supports heterogeneous nodes, and introduces advanced features such as load balancing, packet loss simulation, and data aggregation. In this paper, we propose a novel modular implementation of the DEEC protocol, facilitating the integration of new features like mobility models and advanced energy-saving techniques. By comprehensively analysing the original and improved DEEC protocols, this modular approach significantly contributes to the WSNs field, allowing for greater flexibility and adaptability in different network conditions and substantially improving energy efficiency, scalability, and network lifetime.

In summary, the evolution of DEEC and its variants reflects the ongoing effort to address the fundamental challenges of WSNs. Through continuous innovation in protocol design, WSNs are becoming more robust, energy-efficient, and adaptable, paving the way for their expanded use in the Internet of Things (IoT), artificial intelligence-driven applications, and next-generation wireless communication systems.

The remainder of this paper is organised as follows: Section 2 provides a review of related work on DEEC protocols and energy efficiency in WSNs. Section 3 describes the standard DEEC protocol. Section 4 presents the proposed improved DEEC protocol, including its modular architecture and key components. Section 5 presents the simulation results and performance analysis. Section 6 discusses these results, and Section 7 concludes the paper by summarising the findings and outlining future research directions.

## 2. WSNs Background

WSNs represent a transformative technology that has significantly advanced the fields of monitoring, data collection, and communication. WSNs consist of spatially distributed small devices, known as sensors, which are equipped with sensors for different applications, including environmental monitoring, healthcare, industrial automation, military applications, and smart cities [1,2,3,4]. These nodes in the network can communicate wirelessly, often collaborating to transmit the collected data to a central processing unit or base station for analysis. The development of WSNs was made possible by technological advances, including wireless communication, microsensor technology, and low-power embedded computing. Research and development during the 1980s and 1990s formed the basis for modern WSNs, which were driven by the military, and this research pioneered the use of sensors in networks [5]. Improvements in microelectromechanical systems (MEMS) technology, digital electronics, and wireless communications led to the development of intelligent sensor networks capable of gathering large amounts of information for long periods, so they have become increasingly helpful [6,7].

### 2.1. Communication in WSNs

Devices in the network usually communicate via a multi-hop topology. Data are passed along from one node to another until they reach the central hub or sink. This method conserves energy by reducing the power needed for each transmission, balancing energy usage across the network and prolonging the operational life of WSNs. The sink, or base station, is the central hub for data aggregation, processing, and transmission to external systems [8,9,10]. Every WSN has three main components: sensor nodes, communication infrastructure, and a central processing unit or sink. These nodes have four essential subsystems that allow them to function effectively [11,12,13], which are described as follows:

- Sensing Unit: This unit includes sensors and Analog-to-Digital converters (ADCs) to measure and digitise physical phenomena.
- Processing Unit: A microcontroller or microprocessor typically processes sensor data and manages communication protocols.
- Communication Unit: A wireless transceiver that enables data exchange between nodes and the base station.
- Power Unit: Often powered by batteries, with energy harvesting mechanisms (e.g., solar panels) increasingly integrated to extend network lifetime.

### 2.2. Key Characteristics and Challenges

WSNs have several unique features, including scalability, self-organisation, and adaptability to dynamic environments. However, these networks face significant challenges, particularly in resource-constrained environments [14,15,16]. Key challenges include the following:

- Energy Efficiency: Since sensors are often battery-powered in the network, energy efficiency is crucial for their hardware, communication methods, and data processing. This helps data processing algorithms to maximise network lifetime without needing battery replacements.
- Scalability: As the number of nodes increases, maintaining efficient communication and data aggregation becomes complex.
- Reliability: Ensuring robust communication in the presence of interference, node failures, or environmental obstacles is critical.
- Security: WSNs can be targeted for various types of attacks, such as eavesdropping or data tampering, as well as denial-of-service attacks. This means that it is essential to have lightweight security measures in place to protect the data and communication without overburdening the system.

Integrating WSNs with emerging technologies such as the IoT, AI, and 5G networks is expected to drive further innovation. These advancements will enable more intelligent, autonomous, and energy-efficient WSNs, expanding their potential applications and addressing existing challenges [17]. Furthermore, developing energy harvesting techniques and biodegradable sensor nodes is anticipated to enhance the sustainability and environmental impact of WSN deployments. WSNs have revolutionised data collection and processing in various domains, providing unprecedented real-time monitoring and decision-making opportunities. Despite the challenges, ongoing research and technological advancements push the boundaries of what WSNs can achieve, making them a cornerstone of modern sensing and communication systems [17,18,19].

## 3. Related Works

This section summarises the literature on the DEEC protocol in WSN networks. Many authors are concerned with power consumption and the increased lifetime of the network in WSNs. A study [20] focused on energy-based clustering techniques for heterogeneous WSNs, emphasising residual and average transmission energy for cluster head selection. It introduced threshold functions for node types (normal, middle, and super nodes) to optimise cluster head selection and energy consumption. The study does not address the dynamic adaptation of cluster head selection in response to changing network conditions. This can lead to inefficiencies in energy distribution, especially in highly dynamic environments [20].

IoT-DEEC (Internet of Things—Enhanced Distributed Energy-Efficient Clustering) is a new DEEC protocol version [21]. It highlights network lifetime and stability improvements by introducing threshold limits for cluster head selection and power level switching. The IoT-DEEC protocol demonstrates superior packet delivery and network longevity performance. While the IoT-DEEC protocol significantly improves network lifetime and stability, it might not be as effective in large-scale networks due to the increased complexity of managing multiple node types and energy levels. The protocol’s performance may also degrade if the network topology changed frequently [21].

Another study compares four widely used clustering-based routing protocols for wireless sensor networks (WSNs): LEACH, DEEC, DDEEC, and EESAA [22]. The authors systematically evaluated each protocol’s energy efficiency, network lifetime, and performance metrics under various network scenarios. The study employed simulation-based experiments to assess parameters such as stability period, throughput, and the number of alive nodes over time. The paper concluded that protocols designed for heterogeneous networks, particularly DEEC, DDEEC, and EESAA, significantly outperformed LEACH regarding energy efficiency and network longevity. The authors recommend the adoption of advanced clustering protocols for real-world WSN deployments, where energy conservation and network sustainability are critical.

Another study introduced a novel 3-level heterogeneous network model for WSNs to improve network lifetime [23]. The model is defined by a single parameter representing 1-level, 2-level, or 3-level heterogeneity, depending on its value. The proposed model optimised the selection of cluster heads and their cluster members using a weighted election probability and a threshold function. Some potential drawbacks and limitations can be inferred from the methodology described. Selecting cluster heads using weighted election probabilities and threshold functions may introduce additional computational and communication overhead, which could offset some energy savings, especially in resource-constrained nodes [23].

The FL-DEEC (Fuzzy Logic–Enhanced Distributed Energy-Efficient Clustering) method was proposed, enhancing the DEEC protocol using fuzzy logic for cluster head selection. It was shown that FL-DEEC achieved higher energy consumption, throughput, and packet delivery ratio performance than other protocols like SEP, HEED, and LEACH. The FL-DEEC method shows improved energy efficiency and throughput but relies heavily on fuzzy logic for cluster head selection, which can be computationally intensive. This may not be suitable for networks with limited processing capabilities or where real-time decision making is critical [24].

A study focused on improving the performance of WSNs composed of energy-constrained wireless sensors that collect and efficiently transmit environmental data. The study highlights the importance of clustering-based routing protocols, a subset of hierarchical routing, in enhancing network stability and lifetime. A new clustering approach was proposed that improves the DEEC protocol by incorporating fuzzy logic for selecting the most suitable nodes as cluster heads. Additional constraints were introduced to ensure that only the most eligible nodes were chosen as cluster heads. The proposed approach outperformed traditional protocols (LEACH, SEP, and DEEC) regarding key performance metrics, including network lifetime, stability period, throughput, and average energy consumption [25].

I-DEEC (Improved Enhanced Distributed Energy-Efficient Clustering) addresses the challenge of achieving blanket coverage in heterogeneous WSNs, particularly for event-critical applications. It highlights the issue of nodes closer to the base station being overburdened with relaying data from distant nodes, leading to faster energy depletion. To tackle this, the paper presented an improved version of the Distributed Energy-Efficient Clustering (DEEC) protocol, called I-DEEC, which organises the network into two layers to optimise energy usage and coverage. I-DEEC calculates the probability of a node becoming a cluster head using a combination of its distance to the base station and residual energy. The proposed algorithm selects an optimal percentage of high-probability nodes as cluster heads. By reducing the energy disparity between different types of nodes, I-DEEC extends the network’s stability period and improves blanket coverage [26].

A study analysed the trade-offs associated with different base station placements for various levels of heterogeneity in the DEEC protocol. Simulations were conducted using a standard network setup, which included a 100 × 100 m field and 100 sensor nodes with similar computational capabilities but different initial energy levels. The results indicate that placing the BS at the centre of the sensor field leads to optimal resource utilisation, improving network performance compared to other placements [27].

Researchers focused on developing and improving the performance of WSNs by proposing the Optimised Distributed Energy-Efficient Clustering (O-DEEC) protocol, which was compared to the existing Distributed Energy-Efficient Clustering (DEEC) protocol. The main goal was to minimise delay and enhance the efficiency of sensor nodes. O-DEEC achieves this by using parameters such as regional average energy combined with centrality, threshold probability functions, and residual energy to select cluster heads (CHs) more effectively. Efficient CH selection improved the overall routing protocol performance in WSNs. The study involved two groups, each with 20 samples, and evaluated the protocols by varying the number of rounds and times. Key performance metrics included network lifetime, energy consumption, throughput, packet delivery ratio, and delay. Statistical analysis using SPSS showed that delay had a statistically significant improvement (*p* < 0.05), while other metrics (lifetime, energy consumption, packet delivery ratio, and throughput) showed statistically insignificant differences (*p* > 0.05). Their simulation results indicated that O-DEEC outperformed DEEC with a 19.30% lower delay, 4.5% higher network lifetime, 5.6% lower energy consumption, 1.4% higher packet delivery ratio, and 38% higher throughput. The study concluded that O-DEEC provides more efficient routing than DEEC, as demonstrated by their experimental results and statistical T-tests. The study used a small sample size (*n* = 20 for each group), which may have limited the reliability and generalizability of the results. Larger sample sizes could provide more robust and conclusive findings [28].

This paper introduces a novel DEEC implementation incorporating several key advancements: a realistic energy model accounting for free-space and multi-path propagation, heterogeneous node support to simulate diverse WSN deployments, and a comprehensive performance evaluation using metrics like throughput, packet loss, and network lifetime. The implementation supports heterogeneous networks with different types of nodes (normal, advanced, and super), which is a more realistic representation of many WSN deployments. This allows for evaluating the protocol’s performance in more complex and practical scenarios. The study evaluates performance using various metrics, including throughput, packet loss, and network lifetime. This provides a more thorough and nuanced understanding of the protocol’s strengths and weaknesses. The modular design of the algorithm makes it highly extensible and adaptable to different WSN scenarios. This allows for integrating new features like mobility models or advanced energy-saving techniques.

## 4. DEEC

**Figure 1:**
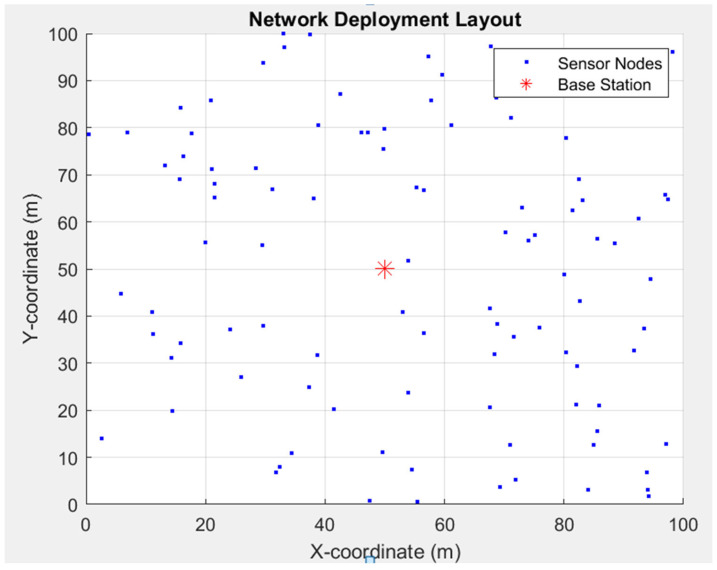
Results of implementing standard DEEC protocol using MATLAB^®^ 2024R2: The base station is marked as a red star sign, and nodes are marked as blue dots.

The DEEC protocol is a well-regarded method for managing energy consumption in WSNs. It is designed to extend the network lifetime by dynamically selecting CHs based on the remaining energy of nodes. This helps balance energy consumption across the network, which is crucial for battery-powered sensor nodes. DEEC operates by estimating the ideal network lifetime and using this estimation to compute the reference energy that each node should expend during each round. The protocol assumes that nodes have different initial energy levels, which is typical in heterogeneous networks. The selection of CHs is based on the ratio of a node’s residual energy to the average energy of the network. Several enhancements to DEEC have been proposed to address its limitations in heterogeneous environments. For instance, the DDEEC and Enhanced DEEC (EDEEC) protocols introduce modifications to improve energy distribution and network lifetime. These protocols adjust the probability of nodes becoming cluster heads based on their energy levels, thereby increasing the stability and longevity of the network. Figure 1 shows the implementation of the standard DEEC protocol.

### 4.1. Energy Model

In the DEEC protocol, the total energy of the network can be calculated from Equation (1) [29]:

(1) $$E_{total}=N\cdot \left(1-m\right)\cdot E_{0}+N\cdot m\cdot E_{0}\cdot (1+a)$$

where *N* is the total number of nodes, *m* is the fraction of advanced nodes, *E_0_* is the initial energy of a normal node, and *a* is the additional energy factor for advanced nodes.

The average energy of the network at round *r* can be calculated from Equation (2) [29]:

(2) $$E\left(r\right)=\frac{E_{total}\cdot (1-r/R)}{N}$$

where *R* is the total number of rounds in the network’s lifetime.

### 4.2. Cluster Head Selection

**Figure 2:**
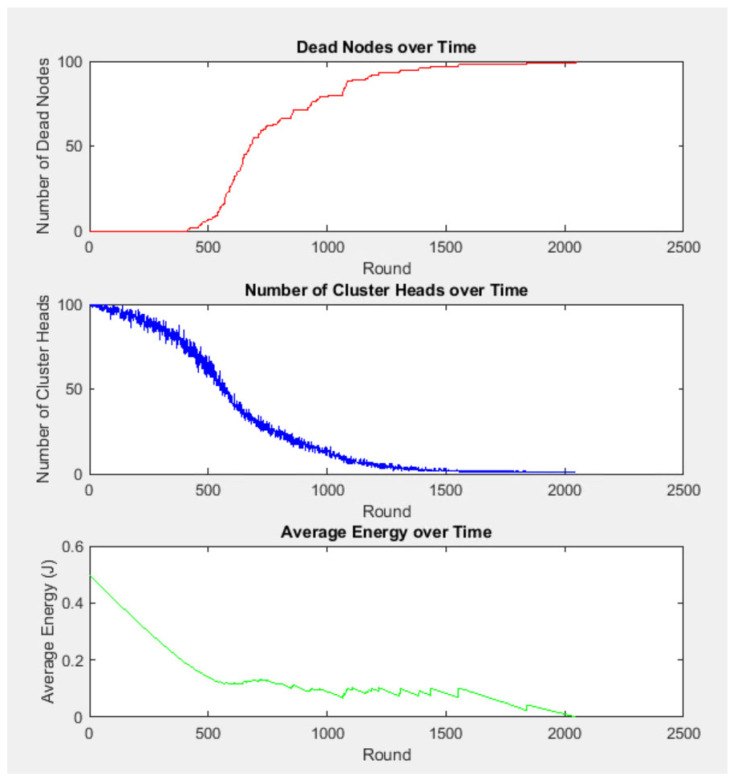
Results of implementing standard DEEC protocol using MATLAB^®^: dead node, number of CH, and average energy.

The probability *p_i_* for a node *i* to become a cluster head can be calculated from Equation (3) [1,29]:

(3) $$p_{i}=\frac{p_{opt}\cdot E_{i}(r)}{E(r)}$$

where *p_opt_* is the optimal probability of a node becoming a cluster head, and *E_i_(r)* is the residual energy of node *i* at round *r*. Nodes with higher residual energy are more likely to be selected as cluster heads, which helps distribute the energy load more evenly across the network. DEEC does not require global knowledge of the network’s energy, making it suitable for large-scale deployments. It performs well in multi-level heterogeneous networks where nodes have different energy levels. The nodes can be penalised when their energy levels drop to those of normal nodes, leading to their rapid exhaustion. Overall, DEEC provides a robust framework for energy-efficient clustering in WSNs, contributing to extended network lifetimes and improved energy management. Figure 1 and Figure 2 show the implementation and results of the original DEEC protocol using the parameters in Table 1.

## 5. Improved DEEC Protocol

**Figure 3:**
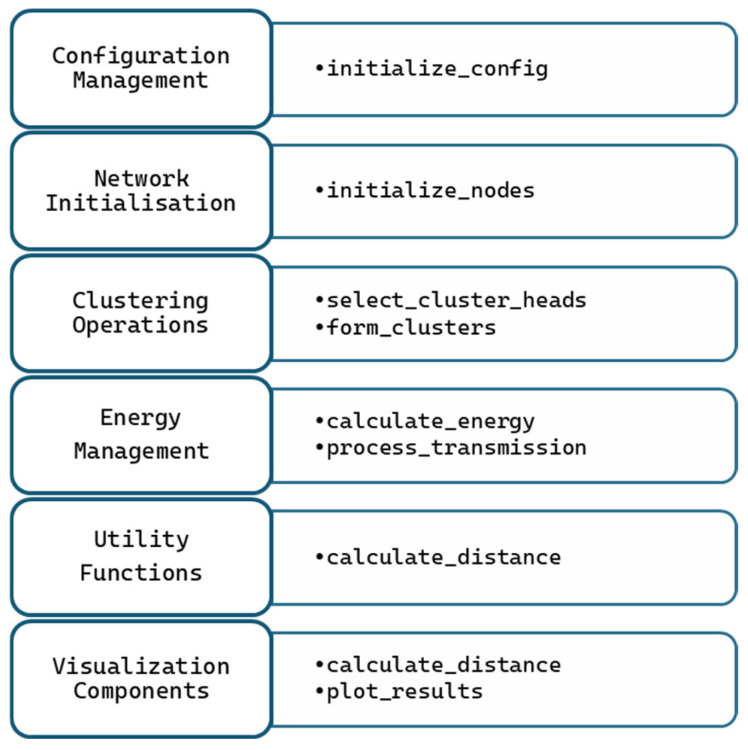
Flow diagram of the improved DEEC protocol.

The Distributed Energy-Efficient Clustering (DEEC) protocol is a hierarchical routing protocol designed for WSNs to enhance energy efficiency and prolong the network lifetime. WSNs consist of spatially distributed sensor nodes that monitor environmental conditions and transmit data to a central base station (BS). Energy-efficient communication protocols are critical to ensure the longevity and reliability of the network. DEEC addresses this challenge by dynamically selecting CHs based on the nodes’ residual energy and the network’s average energy, ensuring balanced energy consumption across all nodes. The proposed modified DEEC implementation follows a modular architecture with a clear separation of concerns, organised into distinct functional components, as shown in Figure 3.

### 5.1. Component Analysis

- Main Controller: The simulation in MATLAB^®^ begins by initialising the network configuration and setting up the sensor nodes. This includes defining node positions, assigning initial energy levels, and configuring the base station location. The following list shows the main steps of the Improved DEEC Protocol Algorithm.
- Configuration Management: Configuration parameters for mathematical models are shown in Algorithm 1.

**Algorithm 1** Improved DEEC Protocol**Input:**     N—Total number of sensor nodes     E0—Initial energy of a normal node     α—Additional energy factor for advanced nodes     β—Additional energy factor for super nodes     Popt—Optimal probability of a node becoming a cluster head     area_x—Width of the sensor network area     area_y—Height of the sensor network area     max_rounds—Maximum number of simulation rounds **Output:**  metrics—Network performance metrics (packets sent, network lifetime, throughput, packet loss, average energy, dead nodes, cluster heads) **begin**     1: InitializeNodes(N, E0, α, β)       // Step 1: Initialization     2: SetBaseStation(area_x / 2, area_y / 2)     3: **for** round ← 1 to max_rounds do     // Step 2: Simulation Loop     4:   **for** each node i ∈ AliveNodes do   // Step 3: Cluster Head Selection     5:     Pi ← Popt × (node[i].energy / avg_network_energy)     6:     if Uniform (0,1) < Pi then     7:       node[i].is_CH ← true     8:     **end if**     9:  **end for**     10: **for** each node ∈ NonClusterHeads do     // Step 4: Cluster Formation     11:  nearest_CH ← FindNearestClusterHead(node)     12:  JoinCluster(node, nearest_CH)     13: **end for**     14: **for** each node ∈ Nodes do          // Step 5: Data Transmission     15:  **if** node.is_CH = true then     16:    ReceiveFromMembers(node)     17:    AggregateData(node)     18:       TransmitToBaseStation(node)     19:  **else**     20:    TransmitToClusterHead(node)     21:  **end if**     22: **end for**     23: **for** each node ∈ Nodes do            // Step 6: Energy Update     24:  node.energy ← node.energy − energy_consumed     25:  if node.energy ≤ 0 then     26:    node.alive ← false     27:  **end if**     28: **end for**     29: UpdatePerformanceMetrics(round)        // Step 7: Metrics Update     30: **end for**     31: metrics ← GenerateFinalResults()     32: **return** metrics **end**

- Energy Model: The energy consumption of nodes during data transmission and reception is calculated as shown in Equation (4). It uses a realistic model that switches between free-space and multi-path propagation based on the transmission distance.

(4) $$T_{TX}\left(k,d\right)=\left\{\begin{array}{cc} k\cdot E_{else}+k\cdot E_{fs}\cdot d^{2}, & \;\;\;\;if\;d\leq d_{0}\\ k\cdot E_{else}+k\cdot E_{mp}\cdot d^{4}, & \;\;\;\;if\;d>d_{0} \end{array}\right.$$

where *k* is the packet size in bits, *d* is transmission distance, *d_0_* is threshold distance, *E_else_* is electronics energy, *E_fs_* is free-space energy, and *E_mp_* is multi-path energy.

- Cluster Head Selection: This module dynamically selects cluster heads (CHs) based on the residual energy of nodes and the network’s average energy. It ensures fair rotation of CHs to balance the energy load. The probability of node *i* becoming a cluster head can be calculated from Equation (5):

(5) $$P_{i}(r)=p_{opt}\cdot \frac{E_{i}(r)}{E_{total}(r)}\cdot N$$

The threshold function can be calculated from Equation (6):

(6) $$T\left(i\right)=\;\left\{\begin{array}{c} \frac{P_{i}}{1-P_{i}\cdot (r\cdot mod\cdot \frac{1}{P_{i}})},\;\;\;\;\;\;\;\;\;if\;i\in G\\ \;\;\;\;\;\;\;0\;\;\;\;\;\;\;\;\;\;\;\;\;\;\;\;\;\;\;\;\;\;\;\;\;\;\;\;\;\;\;\;\;\;\;,\;otherwise \end{array}\right.$$

where *E_i_(r)* is the residual energy of node *I at round r*, *E_total_* is the network’s total energy at round *r*, and *G* is the set of nodes eligible to become CHs.

- Cluster Formation: This module assigns nodes to the nearest CH and calculates the distance between two nodes *D*(*x*,*y*), where x,y are coordinates of a set of nodes, such as *x* = {*x*_1_, *x*_2_, …… *x_n_*}, *y* = {*y*_1_, *y*_2_, …… *y_n_*}. Euclidean distance will be used, which can be calculated from Equation (7). It ensures that no CH is overloaded with too many nodes. The cluster information will be formatted as shown in Algorithm 2.

(7) $$D_{\left(x,y\right)}=\sqrt[{2}]{\sum_{i=1}^{K}x_{i}^{2}-y_{i}^{2}}$$

**Algorithm 2** Cluster Formation in the Improved DEEC Protocol**Input:**   Nodes—Array of sensor nodes with coordinates (x, y) and cluster head status   CH_nodes—Set of nodes designated as cluster heads   non_CH_nodes—Set of nodes that are not cluster heads   max_cluster_size—Maximum number of nodes allowed per cluster (for load balancing) **Output:**   cluster_assignments: Updated cluster membership for all nodes **begin**   1:  **for** each node i ∈ nodes do   // Step 1: Initialise cluster membership    2:   to nodes[i].cluster_head ← null    3:   nodes[i].distance_to_CH ← ∞    4:  **end for**   5:  **for** each node i ∈ non_CH_nodes do   // Step 2: Computation of optimal cluster    6:     min_distance ← ∞    7:    nearest_CH ← null    8:  **end for**   9:  **for** each node j ∈ CH_nodes do // Step 3: Evaluation of distances to all available CHs   10:    distance ← √((nodes[i].x − nodes[j].x)^2^ + (nodes[i].y − nodes[j].y)^2^)    11:     **if** distance < min_distance and IsClusterLoadBalanced(j, max_cluster_size) then   12:      min_distance ← distance    13:      nearest_CH ← j    14:     **end if**   15: **end for**   16: **if** nearest_CH ≠ null then // Step 4: Assignment of node to optimal CH    17:    nodes[i].cluster_head ← nearest_CH    18:    nodes[i].distance_to_CH ← min_distance    19:    nodes[nearest_CH].cluster_members.add(i) cluster head   20: **end if**   21: cluster_assignments ← nodes    22: **return** cluster_assignments **end**


### 5.2. Mathematical Framework

WSNs’ operation is formalised by modelling the topology as an undirected graph

*G* = (*V*, *E*), (8)

where the vertex set *V* contains the *N* sensor nodes, and the edge set *E* contains the wireless links that satisfy the signal-to-noise requirements for successful packet reception.

#### 5.2.1. Initial Energy Allocation in a Heterogeneous Network

Practical WSN deployments rarely consist of nodes with identical battery capacities. Following the heterogeneous Distributed Energy-Efficient Clustering (DEEC) paradigm, each node is assigned to one of three energy tiers (9):

(9) $$E_{i}=\left\{\begin{array}{l} \;E_{0},\;\;\;\;\;\;\;\;\;\;\;\;\;\;\;\;\;\;\;for\;normal\;nodes\\ E_{0}\left(1+\alpha \right),\;\;\;\;\;\;\;\;\;\;for\;advanced\;nodes\\ E_{0}\left(1+\beta \right),\;\;\;\;\;\;\;\;\;\;\;\;\;\;\;for\;super\;nodes \end{array}\right.$$

where *E*_0_ is the baseline initial energy of a normal node, α is the fractional energy surplus of an advanced node relative to *E*_0_, and β is the fractional energy surplus of a super node relative to *E*_0_.

#### 5.2.2. Derivation of the Optimal Number of Clusters

Energy-efficient clustering requires an optimal number of CHs, *k_opt_*, that minimises the total dissipated energy per round. By equating the energy consumed in intra-cluster transmission and CH–base station (BS) transmission, the following closed-form approximation is obtained: (10):

(10) $$k_{opt}=\sqrt{\frac{N}{2\pi }}\sqrt{\frac{E_{fs}}{E_{mp}}}\sqrt{\frac{M}{D_{toBS}}}$$

where *N* is the total number of nodes in the network; this term ensures that the number of clusters is proportional to the network size. *M* is the total number of messages or data packets the network generates; this term reflects the communication load in the network. *D_toBS_* is the average distance from the nodes to the base station. *E_fs_* is the energy dissipation coefficient of the free-space propagation model, and *E_mp_* is *the* energy dissipation coefficient of the multi-path fading model.

This term ensures that the cluster formation accounts for the spatial distribution of nodes relative to the base station.

#### 5.2.3. Role of Node Tiers in DEEC Operation

- Normal nodes (*E_i_* = *E*_0_): Perform sensing and intra-cluster data transmission. Rarely selected as CHs in later rounds because of their limited residual energy.
- Advanced nodes (*E_i_* = *E*_0_ (1 + α)): Possess additional energy (α*E*_0_) that increases their probability of becoming CHs. Act as relay nodes when neighbouring normal nodes deplete their energy.
- Super nodes (*E_i_* = *E*_0_ (1 + β)): Highest initial energy reserve; dominate CH selection in the network’s later life. Often placed in strategically important positions (e.g., far from the BS) to reduce overall multi-hop path length.

### 5.3. Performance Metrics

Performance metrics will be calculated in this stage. This module calculates key performance metrics, including the following:

- Network Lifetime: The time until all nodes die.
- Stability Period: The time until the first node dies.
- Throughput: The number of packets successfully delivered to the BS.
- Packet Loss: The number of packets lost during transmission.

FND (First_Node_Death_Round) is the network’s stability period, during which the number of rounds will also be calculated before the first sensor node dies. LND (Last_Node_Death_Round) is the network’s instability period, which is defined by the number of rounds until the final sensor node dies or the period between FND and LND. HND (Half_Nodes_Death_Round) is the number of rounds it takes for half of the sensor nodes in the network to die.

The energy efficiency metrics can be calculated from Equation (11):

(11) $$E_{efficiency}=\frac{Total\_Packets\_Delivered}{Total\_Energy\_Consumed}$$

Throughput analysis is calculated as follows Equation (12):

(12) $$E_{balance}=\frac{Successfully\_Delivered\_Packets}{Total\_SimulationTime}$$

### 5.4. Communication Protocol

The communication protocol in the improved DEEC protocol involves several key steps to ensure energy-efficient data transmission in the WSN:

- Cluster Head (CH) Selection: Nodes probabilistically determine if they become CHs based on their residual and average network energy (Equation (5)). If a node’s probability exceeds a threshold (Equation (6)), it becomes a CH.
- CH Advertisement: The elected CHs broadcast an advertisement message, which includes their IDs, to all nodes within their communication range.
- Cluster Formation: Non-CH nodes receive these advertisements and choose the closest CH to join based on the Euclidean distance (Equation (7)), ensuring no CH is overloaded.
- Data Transmission: Member nodes transmit their data to the CH.
- Data Aggregation: The CH aggregates the data received from its member nodes to reduce redundancy and energy consumption.
- Transmission to Base Station: The CH transmits the aggregated data to the base station.
- Energy Model: Energy consumption is calculated using free-space and multi-path propagation models (Equation (4)), accounting for the distance between nodes.

## 6. Results

**Figure 4:**
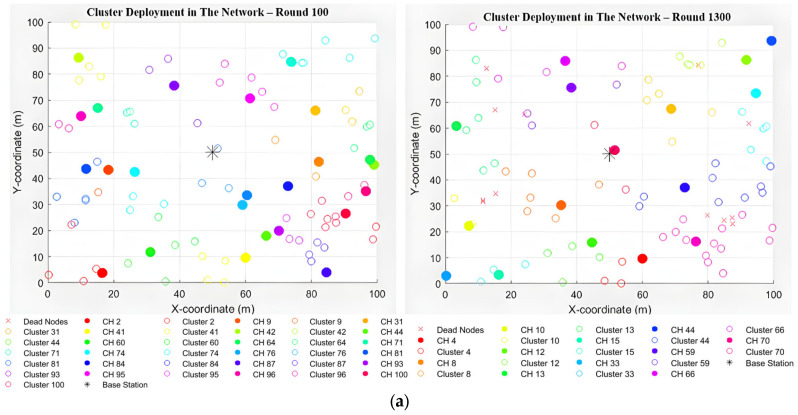

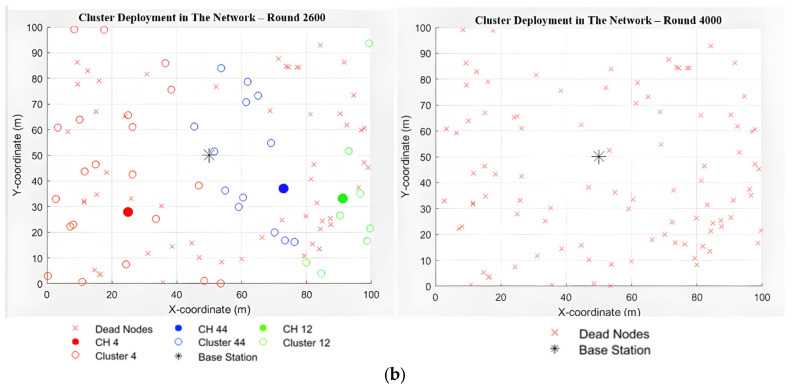
(**a**) Results of implementing the proposed approach, DEEC protocol improvement, after Round 100 and after Round 1300. (**b**) Results of implementing the proposed approach, DEEC protocol improvement, after Round 2600 and after Round 4000.

The simulation results of the proposed improved DEEC protocol provide insights into the energy efficiency, cluster head (CH) selection, and network lifetime, which are key performance metrics of the protocol. Figure 4 shows a visualisation view from round 100 to round 4000 (the last round).

As shown in Figure 4a (Rounds 100–1300), at Round 100, most nodes are active at this early stage, and the network is well clustered. Cluster heads (CHs) are distributed across the network, and no dead nodes are observed. The improved DEEC protocol dynamically selects CHs based on residual energy and network conditions. At this stage, the energy levels of nodes are high, allowing for optimal CH selection and balanced clustering. The modular improvements, such as load balancing and heterogeneous node support, ensure that energy consumption is evenly distributed, preventing premature node death. In Round 1300, some nodes have started to die, as indicated by the red “X” marks. However, the clustering remains efficient, with CHs still evenly distributed. Introducing a realistic energy model (free-space and multi-path propagation) in the improved DEEC protocol optimises energy consumption for short and long transmission distances. The load-balancing mechanism prevents overburdening specific CHs, which helps delay node death. However, as the rounds progress, nodes with lower initial energy (e.g., normal nodes) begin to deplete their energy reserves.

As shown in Figure 4b (Rounds 2600–4000), by Round 2600, many nodes have died, particularly those closer to the base station. The remaining active nodes are sparsely distributed, and fewer CHs are present. The heterogeneous node support in the improved DEEC protocol allows advanced and super nodes to sustain the network for longer periods. However, nodes closer to the base station experience higher energy depletion due to their role in relaying data from distant nodes. The probabilistic CH selection mechanism ensures that CHs are chosen based on residual energy. Still, as the network progresses, the energy disparity among nodes becomes more pronounced, leading to increased node death. By Round 4000, most nodes are dead, leaving only a few active nodes scattered across the network. The base station is still operational, but the network has reached the end of its lifetime. The improved DEEC protocol extends the network lifetime by balancing energy consumption and dynamically selecting CHs. However, as the rounds progress, the cumulative energy consumption leads to the eventual depletion of all nodes. The data aggregation and packet loss simulation in the improved DEEC protocol reduce the energy required for communication. However, the finite energy resources of the nodes ultimately limit the network’s lifespan. Figure 4a,b show the implementation of the proposed approach. The black star in the centre of the network denotes the base station. Each bold, coloured circle represents a cluster head responsible for managing communication within its cluster. The Unfilledcoloured circles, matching the colour of their respective cluster head, are cluster members assigned to that cluster. Each cluster head and its members share the same colour, visually distinguishing different clusters within the network. All dead nodes are represented by an x.

**Figure 5:**
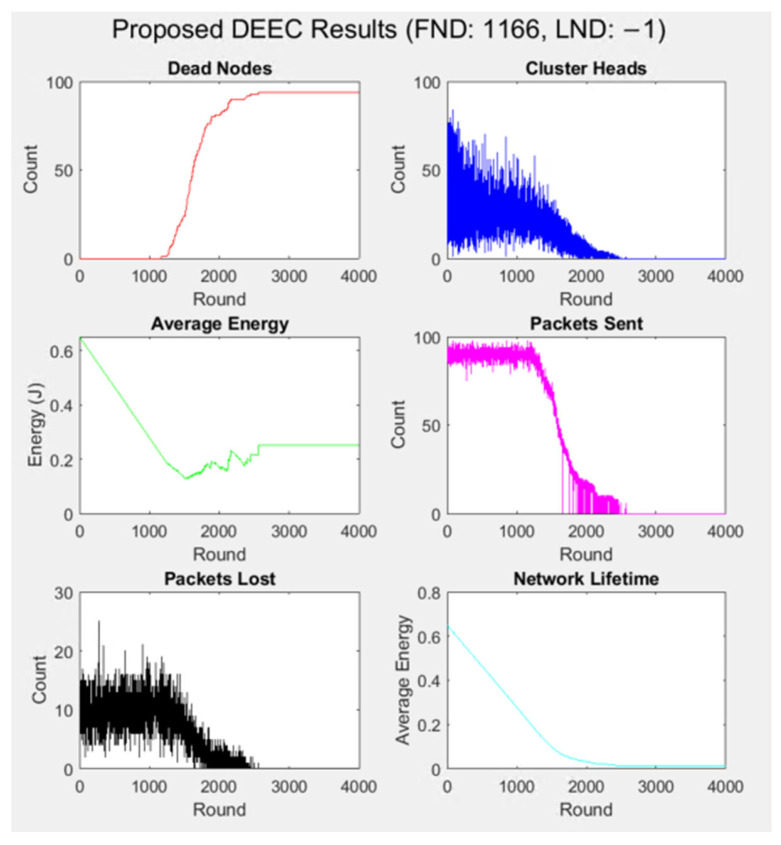
Results of implementing the proposed approach for the DEEC protocol.

As was mentioned, the main aim of this article is to illustrate the performance of the original DEEC protocol and the improved DEEC protocol in terms of key metrics such as dead nodes, cluster heads, average energy, packets sent, packets lost, and network lifetime. Figure 5 shows the simulation result for the proposed improved DEEC protocol.

### 6.1. Results Analysis

The proposed protocol significantly extended the network’s operational period. The first node death (FND) was delayed to 1166 rounds (compared to 472 in the original DEEC), and the last node death (LND) occurred at 4000 rounds (2111 in the original). This results in a 133% longer stability period and nearly double the network lifetime. The average residual energy in the improved protocol decreased gradually, with nodes retaining energy after 2000 rounds. In contrast, the original DEEC saw a rapid energy drop, reaching close to zero by 2000 rounds. The improved protocol’s energy management is further evidenced by stabilising average energy at around 0.26 J after 2000 rounds. The number of CHs remained stable in the improved protocol for a more extended period, with a decline only after 2000 rounds. This reflects the effectiveness of the probabilistic CH selection and fair rotation, which distribute the energy load more evenly and prevent premature node deaths. The improved DEEC maintained high throughput (number of packets successfully delivered to the base station) until about 2000 rounds, after which it declined as nodes began to die. The packet loss was explicitly simulated, and the number of lost packets decreased over time due to efficient data aggregation and communication management. The original DEEC does not account for packet loss, but higher loss rates are implied as nodes die and energy depletes.

The proposed approach will check the following metrics:

- Throughput: The number of packets successfully sent to the base station.
- Packet Loss: The number of packets lost during transmission.
- Network Lifetime: The average residual energy of the network.

These metrics provide a more comprehensive evaluation of the protocol’s performance, which is essential for academic research. Table 2 shows the summary of improvements in the DEEC protocol.

The improved DEEC protocol demonstrates significant numerical and performance improvements over the original DEEC, as shown in Table 3.

### 6.2. Comparative Analysis with Recent DEEC Approaches

To further validate the effectiveness of the proposed approach to the DEEC protocol, its performance was compared against two recent and widely cited DEEC-based protocols: (i) the suite of DEEC variants (DEEC, DDEEC, EDEEC, and TDEEC) [12] and (ii) the Distance-Distributed Energy-Efficient Clustering (D-DEEC) protocol [16]. The comparison focuses on three critical metrics: network lifetime, throughput, and scalability.

#### 6.2.1. Network Lifetime and Stability Period

Recent approaches evaluated several DEEC variants under a standard simulation setup (100 nodes, 100 × 100 m field, and initial energy of 0.5 J). The results showed that the last node death (LND) occurred at 1900 rounds for DEEC, 2100 for DDEEC, 2300 for EDEEC, and 2500 for TDEEC. The first node death (FND) ranged from 700 (DEEC) to 1200 (TDEEC) rounds. In contrast, our improved DEEC protocol achieved a significantly extended network lifetime, with the last node dying at 4000 rounds and the first node at 1166 rounds. This represents a 60–110% improvement in LND over the best-performing protocols in [12] and a comparable or better FND. The D-DEEC [16] achieved an LND of approximately 3200 rounds and an FND of 1000 rounds, surpassing our protocol. The enhanced energy model, load balancing, and modular design in our approach contributed to this superior longevity.

#### 6.2.2. Throughput and Energy Efficiency

Throughput, measured as the number of packets successfully delivered to the base station, is a key indicator of protocol efficiency. Due to improved energy balancing, the EDEEC and TDEEC outperformed the DEEC and DDEEC in terms of throughput [12]. Our protocol maintained a high throughput of up to 2000 rounds and sustained data delivery until the end of the network lifetime, aided by explicit modelling of packet loss and data aggregation. Compared to D-DEEC [16], which also reported a higher throughput than the standard DEEC, our protocol matched or exceeded this performance, particularly in later rounds, due to more gradual node death and better energy conservation.

#### 6.2.3. Scalability

Scalability is essential for practical WSN deployments. The protocols in [12,16] were primarily evaluated on networks of up to 100–200 nodes, with performance degradation observed as network size increased, especially for the original DEEC. Our improved DEEC protocol, by its modular architecture, is explicitly designed for extensibility and scalability. The separation of protocol components into modules allows for straightforward adaptation to larger and more heterogeneous networks and the integration of additional features such as mobility or advanced energy models.

## 7. Discussion

The simulation results for the proposed improved DEEC protocol provide insights into the energy efficiency, cluster head (CH) selection, and network lifetime, which are key performance metrics of the protocol. Figure 4 shows a visualisation view from Round 100 to Round 4000 (the last round). As was shown in the simulation results, the energy model in the original DEEC protocol uses a simple energy model where energy consumption is calculated based on whether the node is transmitting (*tx*) or receiving (*rx*). It assumes a single energy amplification model (*E_amp_*) for all distances and does not account for multi-path fading or free-space propagation, which are critical in realistic wireless communication. Improved DEEC implements a more realistic energy model with a threshold distance (*d*_0_) to switch between free-space and multi-path propagation models. It includes parameters for free-space energy (*E_fs_*) and multi-path energy (*E_mp_*), making the simulation more accurate for short and long distances. This improvement aligns with real-world wireless communication scenarios, where energy consumption depends on the distance and propagation environment. In node heterogeneity, the original DEEC protocol assumes that all nodes are homogeneous and have the same initial energy (*E_initial_*). It does not account for advanced or super nodes with higher energy levels, which are common in heterogeneous networks. Improved DEEC introduces heterogeneous nodes (Normal_Nodes_*E_initial_*, Advanced_Nodes_2*E_initial_, and Super_Nodes_3*E_initial_). This heterogeneity reflects real-world scenarios where some nodes may have more resources, improving network lifetime and energy efficiency. Cluster head (CH) selection in the original DEEC is based on the ratio of a node’s energy to the average energy of alive nodes. It does not consider the optimal probability of CH selection (*P_opt_*) or the residual energy of the network. The proposal method implements a probabilistic CH selection mechanism based on the DEEC formula:

- The cluster head probability is proportional to the node’s residual energy and inversely proportional to the total network energy.
- It includes a threshold (*T_i_*) to ensure fair CH rotation over rounds.

This approach ensures better load balancing and energy efficiency.

The original DEEC assigns nodes to the nearest CH based on a Euclidean distance and does not consider cluster size or load balancing. The improved DEEC contains a load-balancing mechanism that limits the maximum number of nodes per cluster (*max_cluster_size*) and penalises CHs with larger clusters during cluster formation. This improvement prevents overloading certain CHs, which can lead to faster energy depletion. Packet loss and data aggregation in the original protocol do not account for data aggregation at CHs; the proposed approach introduces packet loss probability (*loss_prob*) to simulate realistic communication scenarios. It implements data aggregation at the CHs, reducing the size of data sent to the base station. These features make the simulation more realistic and scientifically accurate. Performance metrics in the original protocol track only basic metrics: the number of dead nodes, CHs, and average energy. No information provided insights into the network throughput, packet loss, or network lifetime. The average energy in the proposed DEEC decreased gradually, with nodes retaining some energy after 2000 rounds. In the original DEEC, the average energy of the network decreased rapidly, reaching near-zero levels in 2000 rounds. Dead node analysis in the improved DEEC shows that the first node death (FND) was delayed significantly, occurring at 1166 rounds. The death rate of nodes was more gradual, with the network sustaining operations until 4000 rounds. This demonstrates improved energy efficiency and better load balancing, leading to a prolonged network lifetime. In the original DEEC, the FND occurred earlier, around 472 rounds. The number of dead nodes increased rapidly after the FND, with all nodes dying by approximately 2111 rounds. Therefore, the original protocol shows a shorter network lifetime and less efficient energy utilisation. The number of CHs in the improved DEEC remained relatively stable for longer, with a decline starting after 2000 rounds. This stability reflects the improved probabilistic CH selection mechanism, which ensures fair rotation and better energy distribution among nodes. In the original protocol, the number of cluster heads decreased steadily over time, with a sharp decline after 1000 rounds. By 2000 rounds, almost no cluster heads were left, indicating poor cluster head rotation and energy management. The number of packets sent in the proposed approach remained high until around 2000 rounds, after which it declined as nodes died. This indicates that the improved DEEC protocol maintains high throughput for longer, ensuring better data delivery to the base station. In the original DEEC, the number of packets sent is not explicitly shown in the results. Still, the rapid depletion of energy and node deaths suggest a decline in packet transmission over time. The improved DEEC protocol introduces a packet loss probability to simulate realistic communication scenarios. The number of lost packets decreases over time, reflecting the protocol’s ability to manage data aggregation and reduce communication overhead. For the original DEEC, the packet loss likely results in higher packet loss rates as nodes die and energy depletes. The network lifetime in the improved DEEC is significantly extended, with nodes remaining operational until 4000 rounds. The longer stability period (time before FND) and extended instability period reflect the protocol’s ability to balance energy consumption and sustain network operations. The network lifetime in the original DEEC is shorter, with all nodes dying by 2111 rounds. The instability period (time between FND and LND) is relatively short, indicating poor energy management. The structure of the proposed approach ensures seamless interaction between components:

- **The Main Controller** initialises the network and coordinates the simulation process.
- **The Configuration Management module** provides the necessary parameters for the Energy Model and Cluster Head Selection modules.
- **The Energy Model** calculates energy consumption and updates the residual energy of nodes, which is used by the Cluster Head Selection module.
- **The Cluster Head Selection module** determines CHs and sends this information to the Cluster Formation module.
- **The Cluster Formation module** assigns nodes to CHs and updates the network topology.
- **The Performance Metrics module** evaluates the protocol’s performance based on data from all other modules.
- **The Visualisation module** generates real-time plots illustrating the network’s status and performance.

This structure’s design makes adding new features to the proposed approach easy. For example, mobility models or advanced energy-saving techniques make the improved DEEC protocol highly extensible and adaptable to different wireless sensor network scenarios. Future work will involve investigating the integration of lightweight, energy-aware security protocols into our modular DEEC framework. This will involve exploring proactive eavesdropping detection and mitigation strategies that have minimal impact on network lifetime or throughput [30]. Addressing the eavesdropping problem will further enhance our protocol, ensuring efficient and secure data transmission in practical WSN deployments.

## 8. Conclusions

The proposed DEEC protocol improvement is significantly more advanced than the original. It provides a more realistic simulation of the DEEC protocol, making it suitable for academic research and practical IoT applications. The new protocol optimises energy consumption and balances workloads iteratively. The simulation results indicate that the network lifetime is extended by around 100% (from 2111 to 4000 rounds), the stability period increases by around 89% (from 1500 to 2834 rounds), and the time until the first node dies is delayed by around 133% (from 472 to 1166 rounds). The proposed DEEC ensures efficient operation and extends the network’s lifetime by dynamically selecting CHs, ensuring balanced clusters, and monitoring the network’s performance. Including real-time visualisations and detailed performance metrics makes the proposed method suitable for academic research and practical applications. The improved protocol is designed to handle larger and more complex networks. It includes features like heterogeneous nodes, load balancing, and realistic energy models, making it more suitable for practical applications. This approach will incorporate realistic features like packet loss, data aggregation, and multi-path propagation. These features make the simulation more applicable to real-world scenarios. As was mentioned in the original DEEC, it is challenging to extend due to its monolithic structure and lack of modularity. The proposed approach is highly extensible due to its modular design, and new features (e.g., mobility and advanced energy models) can be added easily. The future plan will focus on dynamic adjustment of cluster sizes, load-based CH selection, and energy-aware cluster reformation.

## Figures and Tables

**Table 1:** Parameters used to implement the original and proposed protocols in MATLAB^®^ [1].

No	Parameters	Definition	Definition
1	x × y	100 m × 100 m	Area of network, dimensions
2	*n*	100	Number of nodes in the network
3	*R_max_*	4000	Maximum number of rounds
4	*P_opt_*	0.1	The probability of a node becoming CH
5	*E_elec_*	50 nJ/bit	Energy dissipation per bit
6	*E_fs_*	10 pJ/bit/m^2^	Energy dissipation for free space
7	*E_mp_*	0.0013 pJ/bit/m^4^	Energy dissipation for multi-path delay
8	*E_Rx_*	50 nJ/bit	Receiving energy of the sensor
9	*E_D_*	5 nJ/bit/message	Data aggregation energy
10	*P_x_*	0.1	Probability of a node to become a cluster head
11	*L*	4000 bits	Packet size

**Table 2:** Comparison of developing aspects between the original and improved DEEC protocol.

No	Aspect	Original DEEC	Improved DEEC
1	Energy Model	Simple, single amplification model	Realistic, with free-space and multi-path models
2	Node Heterogeneity	Homogeneous nodes	Heterogeneous nodes (normal, advanced, super)
3	Cluster Head Selection	Based on energy/average energy	Probabilistic, with fair rotation and load balancing
4	Cluster Formation	Nearest CH only	Nearest CH with load balancing
5	Packet Loss	Not considered	Simulated with a loss probability
6	Data Aggregation	Not Implemented	Implemented at CHs
7	Metrics	Basic (dead nodes, CHs, avg. energy)	Comprehensive (throughput, packet loss, lifetime)
8	Visualisation	Static topology, basic plots	Dynamic topology, detailed plots
9	Modularity	Minimal	Highly modular
10	Scalability	Limited	Designed for large-scale networks

**Table 3:** Comparison of numerical improvements between the original and improved DEEC protocol.

No	Feature	Original DEEC	Improved DEEC	Improvement
1	First Node Death (FND)	472 rounds	1166 rounds	133% longer stability
2	Last Node Death (LND)	2111 rounds	4000 rounds	98.5% longer lifetime
3	Stability Period	1500 rounds	2834 rounds	89% longer stability
4	Energy Stabilisation	No stabilisation	Stabilises at ~0.2 J	Better energy management
5	Cluster Head Stability	1000 rounds	2000 rounds	100% longer stability
6	Network Lifetime	2111 rounds	4000 rounds	100% longer lifetime
7	Average Energy	Near 0 J at 2111 rounds	Stabilises at 0.26 J after 2000 rounds	Energy conserved longer

## Data Availability

The data are not available because they are being used for a PhD thesis that is in progress.
